# Regulation of *Nir* gene in *Lactobacillus plantarum* WU14 mediated by GlnR

**DOI:** 10.3389/fmicb.2022.983485

**Published:** 2022-10-11

**Authors:** Hulin Qiu, Xiaoyu Chang, Yan Luo, Fengfei Shen, Aiguo Yin, Tingting Miao, Ying Li, Yunyi Xiao, Jinping Hai, Bo Xu

**Affiliations:** ^1^College of Biological and Food Engineering, Guangdong University of Petrochemical Technology, Maoming, Guangdong, China; ^2^Institute for Quality & Safety and Standards of Agricultural Products Research, Jiangxi Academy of Agricultural Sciences, Nanchang, China; ^3^Maoming Branch, Guangdong Laboratory for Lingnan Modern Agriculture, Maoming, Guangdong, China

**Keywords:** *Lactobacillus plantarum*, GlnR, *nir*, bacterial one-hybrid system, qRT-RCR, two-hybrid system, EMSA

## Abstract

Nitrogen (N) is an essential element in the biosynthesis of key cellular components, such as proteins and nucleic acids, in all living organisms. Nitrite, as a form of nitrogen utilization, is the main nutrient for microbial growth. However, nitrite is a potential carcinogen that combines with secondary amines, which are breakdown products of proteins, to produce N-nitroso compounds that are strongly carcinogenic. Nitrite reductase (*Nir*) produced by microorganisms can reduce nitrite. Binding of *GlnR* to the promoter of nitrogen metabolism gene can regulate the expression of *Nir* operon. In this study, nitrite-resistant *Lactobacillus plantarum* WU14 was isolated from Pickles and its protease *Nir* was analyzed. *GlnR*-mediated regulation of *L. plantarum* WU14 *Nir* gene was investigated in this study. New *GlnR* and *Nir* genes were obtained from *L. plantarum* WU14. The regulation effect of *GlnR* on *Nir* gene was examined by gel block test, yeast two-hybrid system, bacterial single hybrid system and qRT-RCR. Detailed analysis showed that *GlnR* ound to the *Nir* promoter region and interacted with *Nir* at low nitrite concentrations, positively regulating the expression of *NIR*. However, the transcription levels of *GlnR* and *Nir* decreased gradually with increasing nitrite concentration. The results of this study improve our understanding of the function of the *Nir* operon regulatory system and serve as the ground for further study of the signal transduction pathway in lactic acid bacteria.

## Introduction

Nitrite and nitrate are the main components of chemical fertilizers. The increased dependence on chemical fertilizers in agricultural production is the main factor that increases soil nitrogen content (Bryan and Grinsven, 2013). Nitrite combines with secondary amines (secondary amines, tertiary amines, amides, and amino acids), the protein decomposition products in fermented food products (pickled vegetables), to form potent cancerogenic nitrosamines in the gastral cavity that are known to cause digestive system cancers (Lin et al., 2013; Dellavalle et al., 2014). In addition, nitrite treatment of horsefish embryos results in yolk sac edema and developmental abnormalities (Simmons et al., 2012; Keshari et al., 2016). Therefore, vegetables with high nitrate content increase the risk of mutagenesis and carcinogenesis in people consuming such fermented food over a long period of time. Moreover, as people become more aware of food safety, they begin to pay more attention to nitrite contamination of fermented foods, and as a result, there is an urgent need to develop measures to degrade the nitrites in fermented food (Sen et al., 2001).

The genus *Lactobacillus* comprises 262 species that are extremely diverse at phenotypic, ecological, and genotypic levels. Zheng et al. (2020) proposed a new taxonomic revision of the *Lactobacillus* genus, reclassifying it into 25 genera which includes host-adapted organisms that have been referred to as the *L*. *delbrueckii* group, *Paralactobacillus* and 23 novel genera. *Lactobacillus planturum*, *Streptococcus lactis*, *Streptococcus thermophilus*, *Lactobacillus casei* and *Lactobacillus brevis* contain high NiR (Zhang et al., 2002; Chi and Zhang, 2017). In 1919, Orla-Jensen confirmed that *L. plantarum* is a facultative hetero-fermentative lactic acid bacterium of the *Streptobacterium plantarum* (Andersson et al., 2005). *L*. *plantarum strain* WU14 was also found to significantly reduce the concentration of nitrites (Ying et al., 2015). Four types of nitrate reductase have been identified, namely cytochrome C nitrite reductase (*nrfA*), nitrite assimilation reductase (*nasB*), copper nitrite reduction enzyme (*nirK*) and cytochrome cd1 nitrite reductase (*nirS*) (He et al., 2015).

LAB are the dominant species during vegetable fermentation. In recent years, the use of pure starter cultures has been shown to be more effective in reducing nitrite concentration compared with spontaneous fermentation (Oh et al., 2004; Yan et al., 2008; Jagannath et al., 2012). Liu et al. (2014) found that *L. casei* were effective in degrading nitrites both in the pickle fermentation system and in MRS medium (Liu et al., 2014). Fang et al. (2016) isolated *Lactobacillus* Coryneformis BBE-H3 from naturally fermented pickles, which showed good nitrite degradation activity (Fang et al., 2016). Therefore, food-grade lactic acid bacteria producing *Nir* can be a safe and effective strategy to dynamically degrade nitrite produced during pickling and deal with nitrite pollution (Liu et al., 2012; Chen et al., 2017).

Genes involved in nitrogen metabolism respond to nitrogen availability in the culture. *GlnR* and *GlnRII*, two regulatory genes, control the expression of several nitrogen metabolism genes at the transcriptional level. Most of the *GlnRs*, including the *GlnR* from Saccharopolyspora erythraea, belong to the OmpR family (Amon et al., 2008). However, the *GlnR* of *Bacillus subtilis* belongs to the *MerR* family (Yao et al., 2014). The OmpR-type response regulator *GlnR* is known to control nitrogen metabolism-related genes. However, it shares no similarity with the *MerR*-type regulator *GlnR* in *B. subtilis* (Brown et al., 2010). *Streptomyces coelicolor* has *GlnR* that regulates nine genes to improve nitrogen transport and metabolic factors in a nitrogen-starved state and conserve cellular resources at high nitrogen levels (Wang et al., 2012). For instance, the *GlnR* of *B. subtilis* acts as a repressor without binding with RNA polymerase (Ogawa et al., 1995). Typically, it binds to target genes in the promoter and controls the transcription of genes related to nitrogen metabolism (Jiang et al., 2014).

*GlnR* activates *nirB* and other genes encoding a putative large subunit of nitrite reductase. In addition, the *GlnR* coactivator *NnaR* binds to the *nirB* promoter (Amin et al., 2012) to promote *nirB* expression. This study analyzed and verified the regulatory mechanism of *Nir* operon in lactic acid bacteria and the effect of transcription regulator *GlnR* on *Nir* expression and the findings contribute to a better understanding of microbial responses to electron acceptor modifications and optimization of nitrate dependent biodegradation strategies.

## Materials and methods

### Strains, vectors, and chemicals

*L. plantarum* strain WU14 was isolated from pickles at the Microorganism Bioengineering Laboratory of the Jiangxi Agricultural University. *Escherichia coli* strains Trans1-T1 and BL 21 (DE3) were obtained from TransGen (Beijing, China). *E. coli* strains were cultured in Luria-Bertani medium (LB; 0.5% yeast extract, 1% tryptone, and 1% NaCl) supplemented with 100 μg/ml kana or AMP. Plasmids T3, *pEASY*-T3 and *pET*-30a were availed for cloning and expression, and the *pGBKT7* and *pGADT7* plasmids were applied in yeast two-hybrid assays (Wang et al., 1995; Guo et al., 2007). The *pB1H1* and *pH3U3* plasmids were used as bait and expression vectors, and the *pSUP202* vector was used to construct the gene knockout plasmids. DNA purification kit, LA Taq DNA polymerase, and restriction endonucleases were sourced from *TaKaRa* (Otsu, Japan), and IPTG was purchased from Sigma (Amresco, United States). All chemicals used in this study were of analytical grade.

### Construction, cloning, and expression of *GlnR* and *Nir* recombinant plasmids

The whole genomic DNA of *L. plantarum* WU14 was extracted by CTAB (Arseneau et al., 2017) method. Primers were designed using DANMAN (Table 1). The PCR product was cloned into the T3 vector and transferred into *E. coli* Trans 1 competent cells for verification and sequencing. Putative transformants were then selected on LB plates containing AMP. The digested T3-*GlnR* and T3-*Nir* plasmids were ligated into the pET-30a (Kang et al., 2019) vector using T4DNA ligase, respectively, followed by transformation into *E. coli* BL21 (DE3) cells and spreading on Kana containing LB plates.

**Table 1:** Primers used in protein expression.

Primers	Sequence	Restriction site
*GlnR* F	5′GGGCGGCCGCCCTAGGATGAAGGAAAAGGAACTC3′	*Not* I
*GlnR* R	5′GGGAAGCTTTTAGTGTGCCGGATAATTGGGGC3′	*Hind* III
*Nir* F	5′GGGCATATGAGTCAAAGCTTATGGCAACGATTG 3′	*Not* I
*Nir* R	5′GGGGCGGCCGCATTCCGTACACTGTTTGCATATTG 3′	Nde I

### *GlnR* and *Nir* protein expression and purification

*E. coli* BL21 competent (Jones et al., 2021) cells harboring recombinant expression plasmids pET-30a-*GlnR* and pET-30a-*Nir* were grown overnight in LB medium containing kana at 37°C in a shaker. The seed culture obtained was inoculated (1%) in LB medium containing AMP and incubated on a shaker at 37°C until OD_600_ reached 0.6 to 0.8. IPTG (Wandrey et al., 2016) was then added to the medium and cultured in the shaker at 30°C for 6 h to induce protein expression. The liquid culture was centrifuged (12,000 g, 10 min, and 4°C), and the collected cells were suspended in 20 ml of NTA-0 buffer. After disruption of the cells by sonication (ice bath), the crude extract was collected by centrifugation (12,000 g, 10 min, and 4°C). The protein in the crude extract was analyzed by SDS-PAGE, purified using nickel column (Ni Sepharose Fast Flow; China), and analyzed again by SDS-PAGE (Scheller et al., 2021).

### Electro mobility shift assay to verify the interaction of *GlnR* and *Nir*

Pnir, the *Nir* promoter, was amplified by PCR with LA-taq and cloned into the T3 vector. *E. coli* Trans 1 competent cells were transformed using this cloned vector, and the putative transformants were confirmed using colony PCR and sequencing. Positive transformants were selected on LB plates (AMP). The digested T3-Pnir fragment and the *GlnR* protein were subjected to a binding reaction. Subsequent to the reaction, the *GlnR*-Pnir complex was analyzed on a 12% polyacrylamide gel (Maizel, 2000).

### Yeast two-hybrid assay

The digested empty vectors *pGBKT7* and *pGADT7* were ligated with *GlnR* and *Nir*, respectively, using the Assemble Mix (Beijing Golden Biological Co., Ltd). The recombinant vectors *pGBKT7*-*GlnR* and *pGADT7*-*Nir* were transformed into Trans1 competent cells and the putative transformants were selected on LB plates containing AMP (Park et al., 2009).

The recombinant plasmid pBD-*GlnR* was transformed into *AH109* yeast competent cells, coated on SD/−Trp/X-α-Gal, SD/−Leu/−Trp/X-α-Gal, and SD/−Leu/−Trp/-His plates and incubated at 30°C for 72 h to detect the toxicity of pBD-*GlnR* protein to the host cells (Lu et al., 2005). Two recombinant vectors *pGBKT7*-*GlnR* and *pGADT7*-*Nir* were co-transfected into the AH109 yeast competent cells with the corresponding empty vectors *pGBKT7* and *pGADT7* as controls and transferred onto SD/−Leu/−Trp/X-α-Gal and SD/−Leu/−Trp/-His+X-α-Gal plates coated with the substrate X-α-Gal for growth analysis (Lu et al., 2005; Song et al., 2018).

### Bacterial one-hybrid assay

#### Construction of pB1H1-*GlnR* bait vector and pH3U3-*Nir* recombinant vector

*GlnR* gene was amplified by PCR, cloned into T3 vector and transformed into Trans 1 competent cells. The transformed cells were screened by colony PCR and sequencing. Positive transformants were selected on LB plates (AMP). The digested T3-*GlnR* recombinant plasmid and the empty vector PB1H1 were ligated with the T4 DNA ligase, followed by transformation into Trans1 competent cells and spreading on LB plates (Amp) for verification. The *pH3U3*/*Nir* recombinant vector was also constructed following the same method (Meng et al., 2005; Xie et al., 2011).

#### Interaction of *GlnR* and *pNir*

The pH3U3-p*Nir* confirmed *via* restriction digestion were electroporated into the constructed US0/pB1H1-*GlnR* competent cells. After PCR of colony, the migration of the different DNAs was assayed *via* gel electrophoresis to verify the results. Finally, US0/pH3U3, US0/pH3U3-p*Nir* and US1/pH3U3-p*Nir* cultures were diluted; 5 μl of each dilution was placed on the positive and negative sieve medium and incubated at 37°C for 36 h to monitor bacterial growth (Hawkins and Guest, 2017).

### qRT-PCR analysis of *GlnR* and *Nir* under nitrite stress

Activated *L. plantarum* WU14 was inoculated (1%) in a 50 ml fresh MRS medium containing 0 mg/ml, 5 mg/ml, 10 mg/ml, and 15 mg/ml NaNO_2_ and the absorbance (OD_600_) of the culture was measured every hour. Lastly, the growth curve of *L. plantarum* WU14 under different levels of nitrite stress was outlined. The nitrite concentration suitable for the growth of the strain was selected to quantify the regulatory mechanism of *GlnR* on *Nir* under nitrite stress.

Total RNA was extracted (remove DNA midway) from *L. plantarum* WU14 culture using Trizol reagent and reverse transcribed into cDNA using Vazyme HiScriptII1st Strand cDNA Synthesis Kit. Shorter fluorescent quantitative PCR amplification product could ensure amplification efficiencies close to 100% for each round. When measured with a real-time PCR instrument, the optimum assay conditions were to keep the synthesized product fragment at 200 bp. Two pairs of primers were adopted to amplify *GlnR* and *Nir* genes with PCR products of 200 bp and 150 bp. Finally, the fluorescence quantitative PCR analysis was performed using the SYBR Green kit.

### Statistical analysis

All data other than those associated with optimization were statistically analyzed using one-way analysis of variance (ANOVA) and a Tukey–Kramer multiple comparison test.

## Results

### Cloning, expression, and purification of *GlnR* and *Nir*

**Figure 1:**
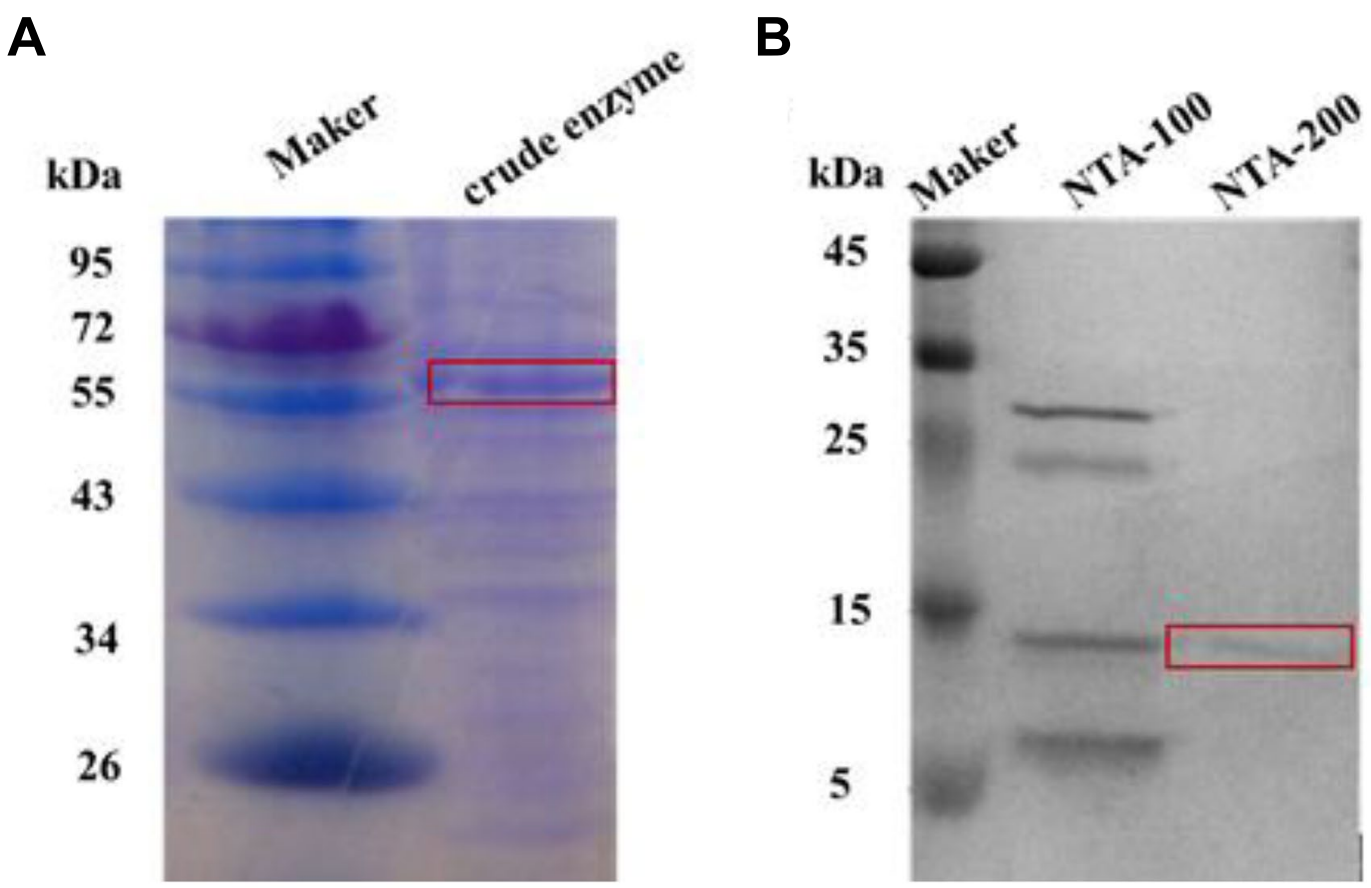
SDS-PAGE analysis of *Nir* and *GlnR*. *Nir* **(A)**; *GlnR* **(B)**.

After successful expression of both proteins in *E. coli*, the crude enzymes were purified by nickel column (Ni Sepharose Fast Flow; China). SDS-PAGE analyses indicated the apparent molecular weights of purified TaBgl1 and IuBgl1 were approximately 62.0 and 14.7 kDa, respectively (Figure 1).

### EMSA indicates the *GlnR* protein-*Nir* interaction *in vitro*

**Figure 2:**
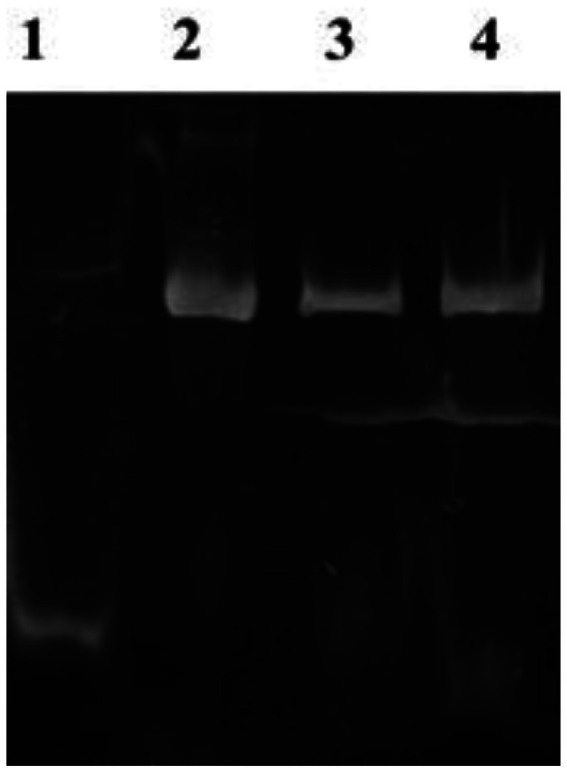
EMSA. Lame 1: *GlnR* protein without *Pnir* gene. Lame 2–4: The complex of *GlnR* protein binding to *NiR* promoter gene.

EMSA was used to assess the interaction between *Nir* DNA and *GlnR* proteins and subsequent regulation of *Nir* transcription. The results suggest that the migration rate of *GlnR* protein, which does not bind to the promoter of nitrite reductase (*Pni*r) on non-denaturing gel, is higher than that of *Pnir*-*GlnR* complex and the band is below the active gel, while the band of *Pnir*-*GlnR* complex is above the band without binding to DNA due to the low mobility (Figure 2). It was shown that *GlnR* protein could recognize the specific sequence above the *NiR* operon, promote the binding of *GlnR* protein with *Pnir*, and therefore, regulate the transcription of *NiR* gene, which confirmed that there are interactions between *GlnR* regulatory protein and *NiR.*

### Yeast two-hybrid assay to investigate the interaction between *GlnR* and *Nir*

**Figure 3:**
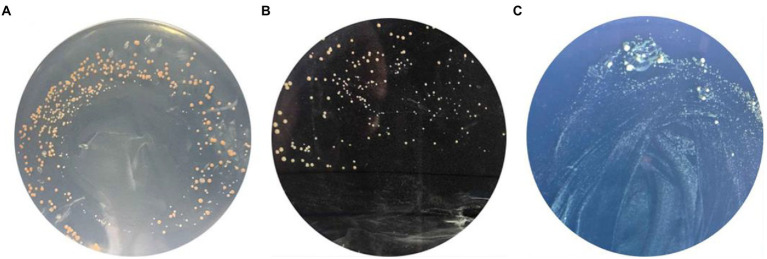
Yeast two-hybrid assay. Self-activation of recombinant vector pBD-*GlnR* **(A)**. The result of empty gsensor conversion of pGBKT7 and pGADT7 **(B)**. The result of interaction of *NiR* and *GlnR* Proteins **(C)**.

**Figure 4:**
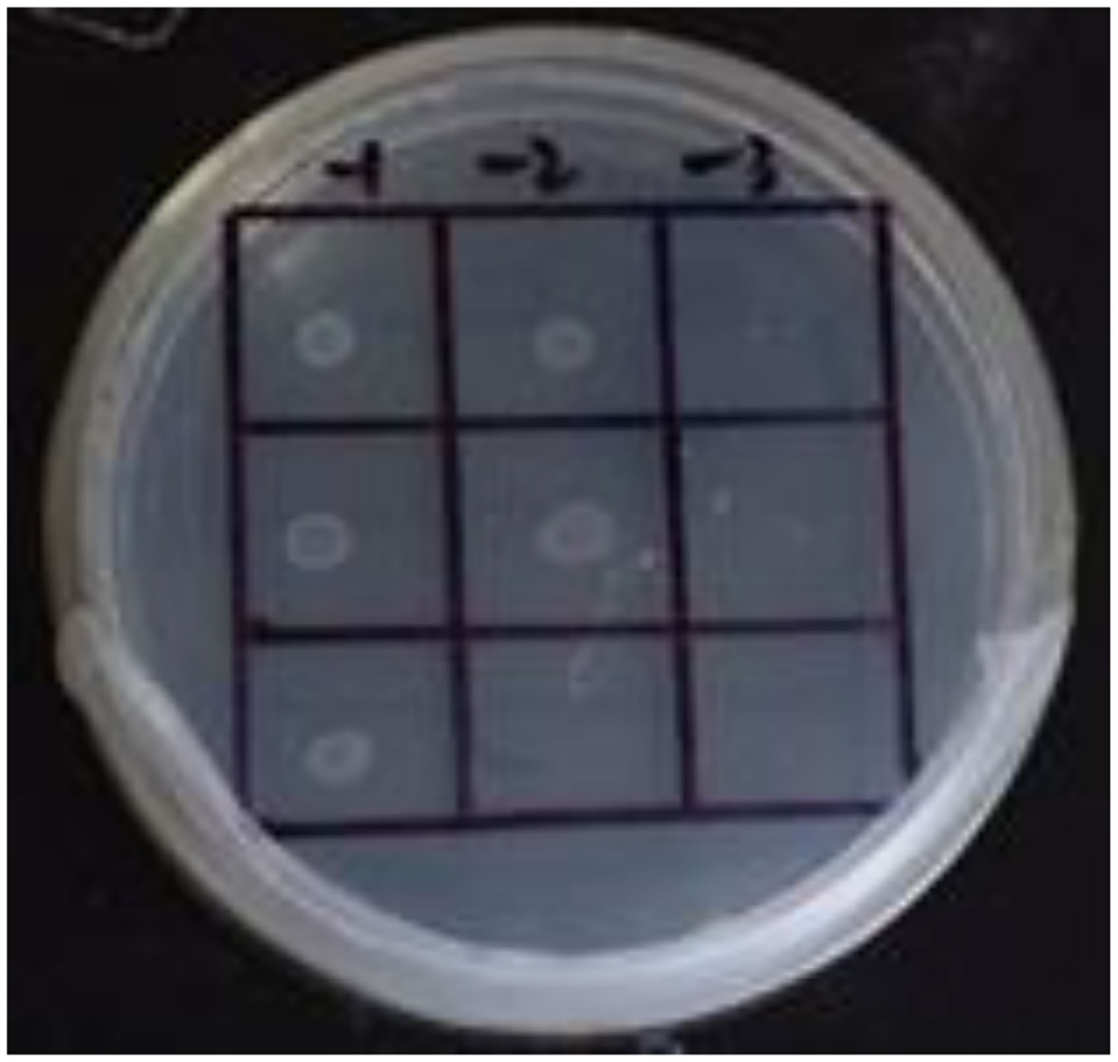
Self-activation of promoter. The first row: US0/pH3U3; the second row: US0/pH3U3-pNir; the second row: US1/pH3U3-pNir. −1, −2 and −3 indicate that the three columns of point samples from left to right are 10^−1^, 10^−2^ and 10^−3^ in the figure of gradually reducing concentrations of the bacterial solution mixed with water.

**Figure 5:**
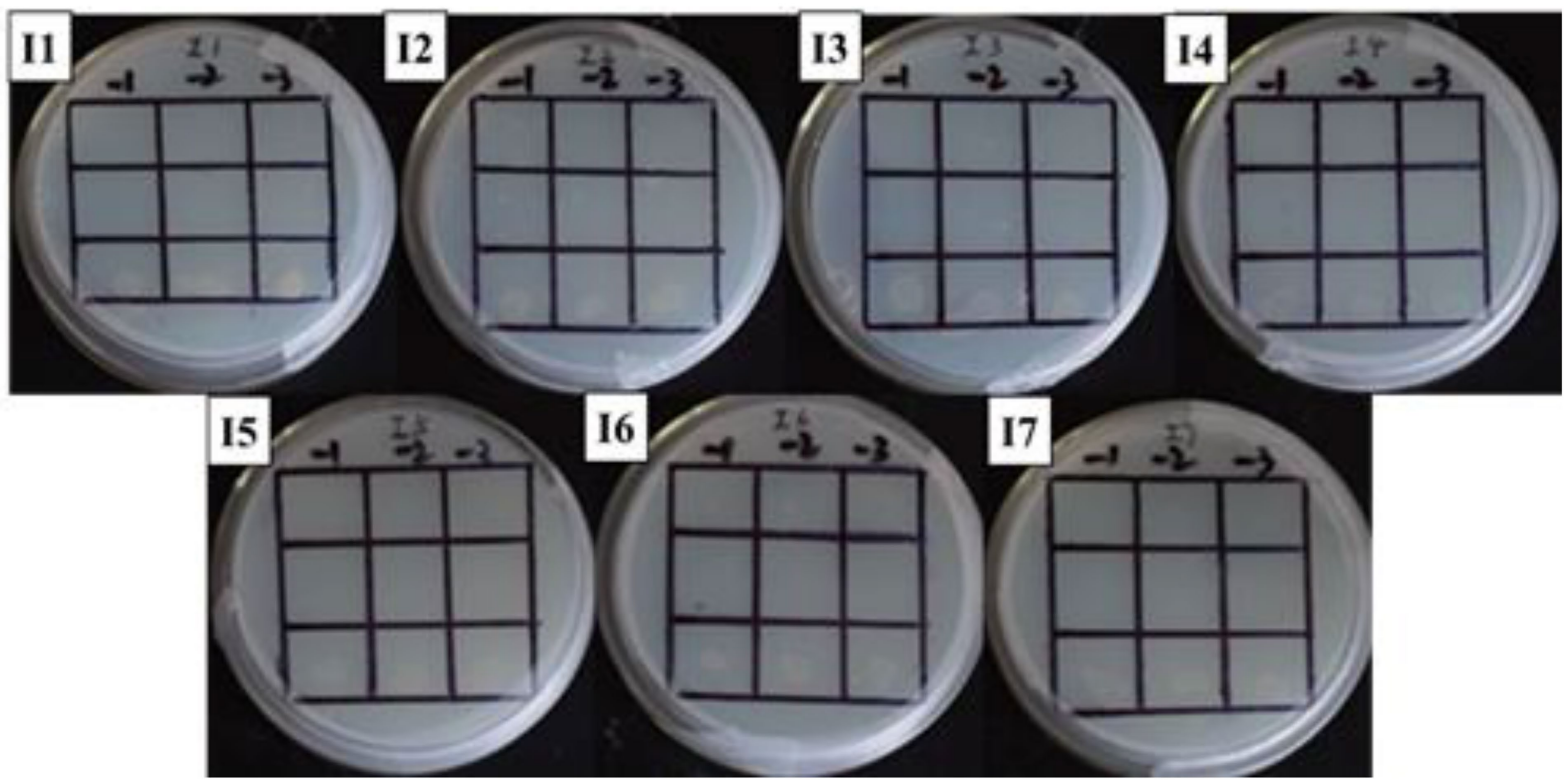
Positive sieve plate with histidine, 0 μg/ml NaNO_2_ (I1), 20 μg/ml NaNO_2_ (I2), 40 μg/ml NaNO_2_ (I3), 60 μg/ml NaNO_2_ (I4), 80 μg/ml NaNO_2_ (I5), 100 μg/ml NaNO_2_ (I6) and 120 μg/ml NaNO_2_ (I7). The first row: US0/pH3U3; the second row: US0/pH3U3-pNir; the second row: US1/pH3U3-pNir. −1, −2, and −3 indicate that the three columns of point samples from left to right are 10^−1^, 10^−2^, and 10^−3^ in the figure of gradually reducing concentrations of bacterial solution mixed with water.

**Figure 6:**
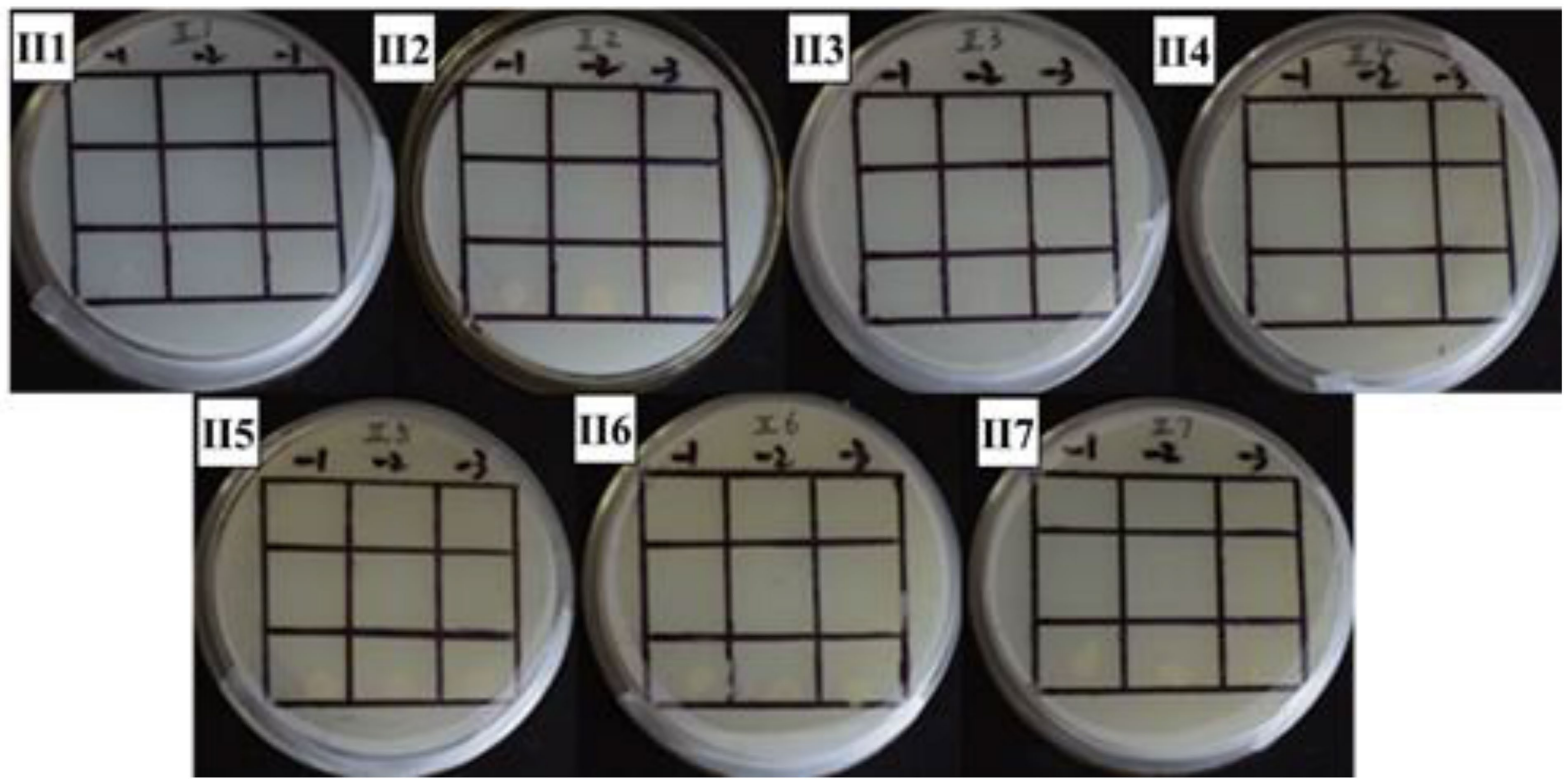
Positive sieve plate with 1 mM 3’AT, 0 μg/ml NaNO_2_ (II1), 20 μg/ml NaNO_2_ (II2), 40 μg/ml NaNO_2_ (II3), and 60 μg/ml NaNO_2_ (II4)80 μg/ml NaNO_2_ (II5), 100 μg/ml NaNO_2_ (II6) and 120 μg/ml NaNO_2_ (II7). The first row: US0/pH3U3; the second row: US0/pH3U3-pNir; the second row: US1/pH3U3-pNir. −1, −2, and −3 indicate that the three columns of point samples from left to right are 10^−1^, 10^−2^ and 10^−3^ in the figure of gradually reducing concentrations of bacterial solution mixed with water.

**Figure 7:**
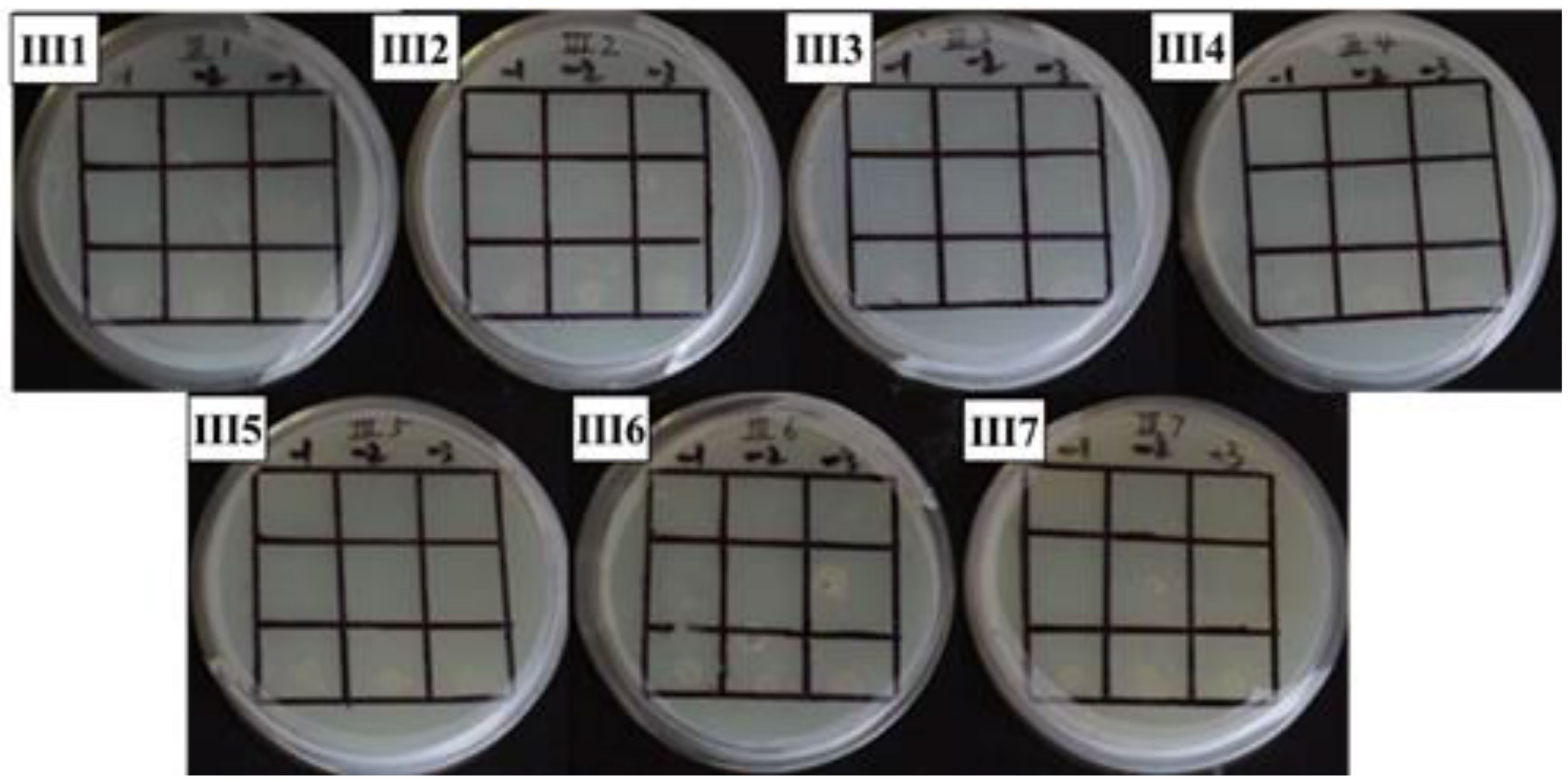
Positive sieve plate with 2 mM 3’AT, 0 μg/ml NaNO_2_ (III1), 20 μg/ml NaNO_2_ (III2), 40 μg/ml NaNO_2_ (III3), 60 μg/ml NaNO_2_ (III4), 80 μg/ml NaNO_2_ (III5), 100 μg/ml NaNO_2_ (III6), and 120 μg/ml NaNO_2_ (III7). The first row: US0/pH3U3; the second row: US0/pH3U3-pNir; the second row: US1/pH3U3-pNir.

Yeast two-hybrid assay was used to study the *GlnR* and *Nir*. In this study, the *GlnR* and *Nir* genes of *L. plantarum* WU14 were cloned, and the bait vector and the reporter vector were constructed; Recombinant plasmid *pBD*-*GlnR* was transformed into AH109 competent cells and coated with SD/-Trp/X-α-Gal double-deficit SD/-Leu/-Trp/X-α-Gal and SD/-Leu/-Trp/-His triple-deficit plates. When the plates were incubated upside down at 30°C for 72 h, pink colonies were found on the missing plate, as shown in Figures 3–7. The results reveal that recombinant vector *pBD*-*GlnR* does not self-activate and the transcription factor GAL4 is not activated, resulting in the expression of downstream galactosidase gene being inhibited and no galactosidase being produced, so the substrate of X-α-Gal on the plate is not degraded and therefore cannot form blue colonies (Figure 3A). Recombinant vectors *pGBKT7*-*GlnR* and *pGADT7*-*NiR* were co-transfected into AH109 yeast competent cells with the corresponding empty vectors *pGBKT7* and *pGADT7* as controls. Pink colonies were observed growing on the second plate of the co-transfected empty vectors *pGBKT7* and *pGADT7* (Figure 3B); however, blue colonies grew on the third plate of the two recombinant plasmids co-transfected (Figure 3C), demonstrating an interaction between *Nir* and *GlnR*. This specific interaction may promote the proximity of the two structural domains (DNA binding domain and BD activation domain), and consequently, initiat transcription of the downstream galactosidase gene in the Y2H assay.

### Bacterial one-hybrid assay

In this study, the *GlnR* gene was inserted into the bait vector to construct the pB1H1-*GlnR*, while the *Nir* gene was inserted into the reporter vector to construct the pH3U3-*pNir*. *E. coli* cells were killed by 5-fluoroorotic acid or failed to survive at low concentrations and grow when ura3 was activated (Figure 4). In the present experiment, the US1/pH3U3-*pNir* strain (the third row) did not grow on the negative sieve medium, which ruled out inaccurate autoactivation. In contrast, the US0/pH3U3 (the first row) and the US0/pH3U3-*pNir* (the second row) strains grew as larger colonies, indicating a lack of ura3 expression in US0/pH3U3 and US0/pH3U3-*pNir* strains. These observations indicate the absence of promoter autoactivation in the negative sieve medium.

The interaction between *GlnR* protein and *pNir* was confirmed in the positive screening medium. On the positive screening medium without sodium nitrite, the first row of US0/pH3U3 and the second row of *US0/pH3U3-pNir* failed to grow, while the third row of strains interacting with target genes generated larger colonies on the positive screening medium (Figure 5, I1). This indicated that the reporter gene his3 was expressed in the positive screening medium for the strain interacting with the protein and the target gene and the strain could grow normally on the His-deficient medium. Similarly, the interaction between *GlnR* protein and *pNir* under nitrite stress was observed in the medium supplemented with appropriate concentration of nitrite (Figures 5–7). Such results are identical to those obtained with positive sieve medium without nitrite. Therefore, the bacterial single hybridization experiment reveals that *GlnR* protein can bind to *pNir* in the cells of US0 strain, and *GlnR* protein can also interact with *pNir* under nitrite stress. In the positive screening medium supplemented with histidine, US0/pH3U3 and US0/pH3U3-*pNir* did not grow properly. The normal growth of US1/pH3U3-*pNir* strain might be caused by the insufficient concentration of histidine in the His-deficient medium (Figures 6, 7). Therefore, the bacterial single hybridization experiments described above demonstrate that *GlnR* protein interacts with *Nir* promoter fragment.

### qRT-PCR analysis of *GlnR* and *Nir* under nitrite stress

The transcript levels of *GlnR* and *Nir* genes significantly increased as the nitrite concentration increased to 1 mg/ml; however, their levels significantly reduced with an increase in the nitrite concentration to 3 mg/ml (Table 2). These observations indicate that under low concentrations of nitrite, *GlnR* positively regulates the *Nir* gene, and the transcript level of the *Nir* gene increase, increasing the amount of nitrite reductase. However, with the gradual increase in nitrite concentration, the transcript levels of *GlnR* and *Nir* genes decrease significantly, which indicates that high nitrite concentration is not conducive to bacterial growth. Furthermore, there is a decrease in the production of GlnR protein and a consequent decrease in the production of nitrite reductase.

**Table 2:** The results of fluorescence quantitative experimental analysis.

Gene	Ct value			Ct average	ΔCt	ΔΔCt relative copy number 2^−△△Ct^
	1	2	3			
*GlnR* (0 mg/ml)	28.32	28.04	28.06	28,0.14	0	1
*GlnR* (1 mg/ml)	28.56	28.7	28.47	28.57	0.07	1.05
*GlnR* (3 mg/ml)	28.58	28.62	28.62	28.67	0.95	0.51
*Nir* (0 mg/ml)	32.51	31.63	31.95	32.03	0	1
*Nir* (1 mg/ml)	31.81	31.7	31.48	31.66	0.87	1.83
*Nir* (3 mg/ml)	32.72	32.47	32.39	32.49	0.88	0.54

## Discussion

There are eight modes of nitrogen cycling in nature, six of which are attributed to microorganisms. Among the six ways, two are the breakdown of nitrite in the presence of nitrite reductase (Kuypers et al., 2018). Namely, denitrification and nitrite ammoniation, with nitrite reductase (*Nir)* being the rate-limiting enzyme among enzymes that catalyze denitrification (Chen et al., 2017). Researchers had identified four types of *Nir*: *nrfA*, *nasB*, *nirK*, *nirS*. Meanwhile, the reduction of nitrite to ammonium was catalyzed by the cytochrome C nitrite reductase encoded by *nrfA* and the nitrite assimilation reductase encoded by *nasB* (Olmo-Mira et al., 2006).

Studies on the biochemical and molecular mechanisms of *Nir* have been mainly focused on denitrifying nitrogen-fixing bacteria such as Pseudomonas stutzeri and Paracoccus Dentifrice. Theoretically, nitrite degradation is involved in two pathways. In the 1980s, the NO−2 degradation pathway was reported in nitrifying bacteria, but the nitrite degradation pathway of the *Lactobacillus* genus is still unclear. However, the molecular mechanism of remains unclear (Liu et al., 2014; Paik and Lee, 2014) as there are few studies reporting the structure, function and regulatory mechanism of *Nir* operon and the transcription regulatory protein of lactic acid bacteria.

*GlnR* of *S. coelicolor* is known to activate or inhibit ten target genes in the operon responsible for nitrogen absorption and regulation (*amtB*-*glnK*-*glnD*) and encoding the nitrite and nitrate degradation enzymes (*nirB* and *nasA*), urease (*ureA*), and glutamate and glutamine synthetase (*glnA*, *glnII*, and *gdhA*) (Tiffert et al., 2008; Sola et al., 2013). *GlnR* is a transcriptional regulator widely distributed in microorganisms and it has been divided into the *MerR* family and the OmpR family (Ying et al., 2015). It has been found that *GlnR* regulators are instrumental in the regulation of nitrogen metabolism. Many nitrogen metabolism-related genes, such as *gdhA*, *glnPQ*, *amtB*, *nirBD*, *ureA*, *glnK* and *glnD* are regulated by *GlnR*. *GlnR* is a transcriptional regulator of nitrogen metabolism genes, such as *glnA* (Arcondéguy et al., 2001; Amon et al., 2008; He et al., 2015).

The *GlnR* of lactic acid bacterial strain *Lactococcus lactis* MG1363 also possesses ten target genes, including the direct target *glnRA* operon, the ammonium transport and sensor operon amtB-*glnK*, and the glutamine/glutamate ABC transporter encoding gene *glnPQ* (Larsen et al., 2006). However, based on the protein alignment and the predicted three-dimensional structure, *GlnR* of *L. plantarum* WU14 belongs to the *MerR* family, which is consistent with the *GlnR* in of *B. subtilis* (Fisher, 1999). Typically, transcriptional proteins of *MerR* family adopts a helix - aAngle - helix model, similar to that of *GlnR*. The most prominent features of *MerR* proteins are their conserved sequences at the N-terminal and the specific sequences at the C-terminal, which are also reflected in *GlnR* (Helmann et al., 1990).

In this study, the structure and function of *GlnR*, a transcriptional regulator of *L. plantarum* W14 were analyzed, and accordingly, it was found to induce the expression of *Nir* and *GlNR*-mediated regulation of nitrogen metabolism in food-grade plant lactic acid bacteria cells. The interaction between *GlnR* and the *NIR* operon and promoter in nitrogen metabolism of *L. plantarum* W14 was demonstrated by bacterial single hybrid experiments, which preliminarily confirmed a major contribution of *GlnR* in the positive regulation of *NIR* gene transcription in *L. plantarum* WU14 at low nitrite concentrations. The transcriptional levels of *GlnR* and *Nir* genes at different concentrations of nitrogen sources were analyzed by fluorescence quantitative PCR. The results demonstrate that *GlnR* is involved not only in ammonium assimilation, but also in ammonium supply. In the absence of *GlnR* gene, even nitrate-providing bacteria cannot grow and the expression of at least one gene required for nitrate reduction is compromised. *GlnR* targets in *L. plantarum* W14 share similarities with some other Gram-positive bacteria, with a number of differences, though. Further research efforts will target on verifying the importance of *GlnR* in the regulation of nitrogen metabolism in *L. plantarum* and finding new targets of *GlnR* in *Lactobacillus* brevi. In addition, the specific mechanism of *Nir* gene regulation in *L. plantarum* WU14 are yet to be investigated.

## Data availability statement

The original contributions presented in the study are included in the article/supplementary material, further inquiries can be directed to the corresponding author.

## Author contributions

HQ: conducted experiments and wrote the papers. XC: analyzed data. YL: provided technical support. FS: proofread the papers. BX: hosted the project and provided financial and technical support. All authors contributed to the article and approved the submitted version.

## Funding

This work was supported by the Guangdong Basic and Applied Basic Research Foundation (2019A1515011696 and 2022A1515012380), and the Guangdong Special Project on Key Fields of Colleges and Universities (Rural Revitalization, 2020ZDZX1020). The funders had no role in study design, data collection and interpretation, or the decision to submit the work for publication.

## Conflict of interest

The authors declare that the research was conducted in the absence of any commercial or financial relationships that could be construed as a potential conflict of interest.

## Publisher’s note

All claims expressed in this article are solely those of the authors and do not necessarily represent those of their affiliated organizations, or those of the publisher, the editors and the reviewers. Any product that may be evaluated in this article, or claim that may be made by its manufacturer, is not guaranteed or endorsed by the publisher.

## Abbreviations

*L. planturum*: *Lactobacillus planturum*
Amp: Ampicillin
Kana: Kanamycin sulfate
IPTG: Isopropyl-β-D-thiogalactopyranoside
SDS-PAGE: Sodium Dodecyl Sulfate-Polyacrylamide Gel Electrophoresis
EMSA: Electrophoretic Mobility Shift Assay
